# Clinical utility of C-reactive protein-based triage for presumptive pulmonary tuberculosis in South African adults

**DOI:** 10.1016/j.jinf.2022.10.041

**Published:** 2023-01

**Authors:** Claire J Calderwood, Byron WP Reeve, Tiffeney Mann, Zaida Palmer, Georgina Nyawo, Hridesh Mishra, Gcobisa Ndlangalavu, Ibrahim Abubakar, Mahdad Noursadeghi, Grant Theron, Rishi K Gupta

**Affiliations:** a Institute for Global Health, University College London, London, UK; b DSI-NRF Centre of Excellence for Biomedical Tuberculosis Research, South African Medical Research Council Centre for Tuberculosis Research, Division of Molecular Biology and Human Genetics, Faculty of Medicine and Health Sciences, Stellenbosch University, Cape Town, South Africa; c Division of Infection and Immunity, University College London, London, UK

**Keywords:** Diagnosis, Screening, CRP, HIV, TB

## Abstract

**Background:**

Identification of an accurate, low-cost triage test for pulmonary TB among people presenting to healthcare facilities is an urgent global research priority. We assessed the diagnostic accuracy and clinical utility of C-reactive protein (CRP) for TB triage among symptomatic adult outpatients, irrespective of HIV status.

**Methods:**

We prospectively enrolled adults reporting at least one (for people with HIV) or two (for people without HIV) symptoms of cough, fever, night sweats, or weight loss at two TB clinics in Cape Town, South Africa. Participants provided sputum for culture and Xpert MTB/RIF Ultra. We evaluated the diagnostic accuracy of CRP (measured using a laboratory-based assay) against a TB-culture reference standard as the area under the receiver operating characteristic curve (AUROC), and sensitivity and specificity at pre-specified thresholds. We assessed clinical utility using decision curve analysis and benchmarked against WHO recommendations.

**Results:**

Of 932 included individuals, 255 (27%) had culture-confirmed pulmonary TB and 389 (42%) were living with HIV. CRP demonstrated an AUROC of 0·80 (95% confidence interval 0·77–0·83), with sensitivity 93% (89–95%) and specificity 54% (50–58%) using a primary cut-off of ≥10 mg/L. Performance was similar among people with HIV to those without. In decision curve analysis, CRP-based triage offered greater clinical utility than confirmatory testing for all up to a number willing to test threshold of 20 confirmatory tests per true positive pulmonary TB case diagnosed (threshold probability 5%). If it is possible to perform more confirmatory tests than this, a ‘confirmatory test for all’ strategy performed better.

**Conclusions:**

CRP achieved the WHO-defined sensitivity, but not specificity, targets for a triage test for pulmonary TB and showed evidence of clinical utility among symptomatic outpatients, irrespective of HIV status.

**Funding:**

South African Medical Research Council, EDCTP2, Royal Society Newton Advanced Fellowship, Wellcome Trust, National Institute of Health Research, Royal College of Physicians.

## Highlights

• CRP has good diagnostic accuracy for pulmonary TB among symptomatic adults.
• At ≥10 mg/L CRP approaches, but fails to meet, WHO benchmarks for a TB triage test.
• CRP may still offer clinical utility to prioritize use of confirmatory tests.
• Performance is similar across key risk groups for TB including people living with HIV.
• Clinical utility of CRP is dependent on target population TB prevalence.

## Existing evidence

We performed a systematic search using terms for “C-reactive protein” and “tuberculosis” in OVID Medline on 1st April 2021 with no language or date restrictions. Two previous systematic reviews assessed the diagnostic accuracy of C-reactive protein for pulmonary TB, the most recent of which reported pooled sensitivity of 89% (95%CI 80–96%) and specificity 57% (36–65%) using a threshold of ≥10 mg/L. Most included studies were restricted to provider-initiated systematic screening (active case finding) for TB among people living with HIV at the time of ART initiation. One subsequent case-control diagnostic accuracy study found that CRP-based triage did not achieve the WHO target product profile (TPP) criteria for a triage test. An individual participant data meta-analysis evaluated CRP as a screening test for pulmonary TB among PLHIV, irrespective of signs and symptoms, describing 83% sensitivity (95%CI 79–86) and 67% specificity (95%CI 60–73; *n* = 3187) in that setting. Overall, CRP is a promising tool for TB screening and triage, however there are fewer data on the symptomatic triage use case, and no studies have prospectively evaluated CRP-based triage among outpatients presenting with symptoms compatible with TB, without pre-selection based on HIV status or other factors. Moreover, previous data have focused on diagnostic accuracy in isolation, without considering programmatic clinical utility.

## Added value of this study

To our knowledge, this is the first study to report the diagnostic accuracy and clinical utility of CRP for pulmonary TB in a large, consecutive cohort of symptomatic outpatients, irrespective of HIV status. At a threshold of ≥10 mg/L, CRP approached, but did not meet, WHO TPP criteria for a triage test, achieving the minimum required sensitivity (target: 90%; CRP: 93% [95% CI 89–95%]) but not specificity (target: 70%; CRP: 54%;50–58%]). The discriminatory ability of CRP was similar across key risk groups for TB including people living with HIV, and among those with previous TB. In our study population, with very high prevalence of pulmonary TB (27%), a CRP-based triage strategy would lead to 41% fewer confirmatory tests than a test all approach but would miss 7% of TB cases. CRP-based triage demonstrated clinical utility in similar settings if it is necessary or desirable to prioritize use of confirmatory testing so that those tested have at least a 5% risk of pulmonary TB (i.e. a number willing to test of fewer than 20 confirmatory tests per true TB case detected), whilst confirmatory testing for all individuals who met our symptom-based enrolment criteria appears best below this threshold probability. We also demonstrate that clinical utility is highly dependent upon TB prevalence, suggesting that there is likely even more clinical utility for CRP-based triage in settings with lower TB prevalence than that in our study.

## Implications

Previously published data have supported the use of CRP testing to systematically screen for TB among people living with HIV initiating ART. In our study, CRP approaches the WHO benchmarks for a triage test and may offer clinical utility to prioritize use of confirmatory tests among symptomatic adults, without the need for prior knowledge of HIV status. Further urgent prospective evaluations of CRP-guided triage using point-of-care assays, including interventional trials and health-economic analyses, are required to support policy development, and as a benchmark against which to assess the performance of other candidate triage tests.

## Background

Of the estimated 10 million people who developed tuberculosis (TB) in 2020, 4.1 million were not reported to TB programmes.^1^ The World Health Organization (WHO) has emphasised the urgent need for better tools to identify people with TB. A key priority is development of a rapid, point-of-care, low-cost, non-sputum-based “triage” test to guide confirmatory testing. An effective triage test may confer dual benefits in reducing unnecessary confirmatory testing for people at lower risk of disease, while focusing attention on those who need investigations most.^2^^,^^3^ Computer assisted diagnostic radiography and protein or mRNA biomarkers are under active evaluation for this purpose. A triage test prioritises sensitivity over specificity, but to date the desired performance parameters have been informed only by expert opinion and a modeling analysis of potential financial savings. A more holistic framework for evaluating the clinical utility of candidate triage tests has been lacking.

Assuming a triage test threshold is selected to maximize sensitivity, the fundamental question becomes the range of false-positive rates within which a triage test (that triggers confirmatory testing) still achieves a net benefit, compared to confirmatory testing for all. Decision curve analysis (DCA) of test performance in natural observational cohort studies can be used to compare relative net-benefit between two approaches. In DCA, net benefit is calculated as the true positive rate minus the false positive rate weighted by the perceived cost: benefit ratio of an intervention, in this case, confirmatory testing for tuberculosis. The perceived cost: benefit ratio is represented by the “threshold probability” of disease that will trigger an intervention. More intuitively, this is the inverse of the number of confirmatory tests we are willing to undertake to find a true positive TB case. DCA can help us find the number willing to test (NWT) range, where a triage test leads to greater net benefit than confirmatory testing for all.

C-reactive protein (CRP) is an acute phase reactant commonly used as a non-specific marker of inflammation. Proposed use cases in TB have therefore focused on screening and triage rather than confirmatory testing, owing to a lack of specificity. It is measurable on finger-prick blood using point-of-care platforms at a cost of US$2 per test with results in minutes, thus meeting many of the WHO-recommended operational characteristics of a triage test.^2^ Recent WHO guidance has recommended CRP (with a threshold of >5 mg/L) for TB screening among people living with HIV (PLHIV), with a meta-analysis demonstrating 90% sensitivity (95% confidence interval [95%CI] 78–96%) and 50% specificity (29–71%) among outpatients initiating anti-retroviral therapy (ART).^4^ However, the value of CRP as a triage test among symptomatic people presenting to healthcare, including populations with mixed HIV status, is less clear. In two systematic reviews, no identified studies prospectively evaluated CRP-based triage in this context.^5^, ^6^, ^7^, ^8^, ^9^ One subsequent case-control study evaluated the diagnostic accuracy of CRP for pulmonary TB using a threshold ≥12 mg/L and described sensitivity 85% (95%CI 80–88%) and specificity 70% (65–74%). The context in which CRP-based triage may provide clinical utility by reducing the need for confirmatory testing has not been evaluated.^10^

We sought to address these important evidence gaps by testing the diagnostic accuracy and clinical utility of CRP for identifying adult patients with symptomatic tuberculosis in a large prospective observational cohort in South Africa, representative of a setting with high burden of tuberculosis and HIV. In this context, we sought to identify NWT range where CRP achieves greater clinical utility than confirmatory testing for all.

## Methods

### Study population

Consecutive adults (≥18 years) with at least one (for people with HIV) or two (for people without HIV) WHO-defined TB symptoms^4^ presenting to two primary health clinic-based TB services in Cape Town, South Africa were invited to participate (April 2016–November 2020). The study was approved by the Stellenbosch University Faculty of Health Sciences Research Ethics Committee (N14/10/136). All participants provided informed written consent.

### Laboratory procedures

Participants provided two sputum specimens. The first underwent two Ziehl-Neelsen stains, microscopy, and liquid TB culture (MGIT960); the second was tested with Xpert MTB/RIF Ultra (hereafter ‘Ultra’), retrospectively on samples collected prior to its implementation (2016–2017) and through routine care thereafter.^11^ Sputum induction was conducted where required.^12^ Participants not self-disclosing that they were living with HIV were offered HIV testing. CD4 counts and hemoglobin were extracted from clinic records when conducted as part of routine care. We classified anemia per WHO definitions.^13^ CRP was measured in biobanked serum cryopreserved at −80 °C within four hours of collection, using the Cobas high-sensitive immunoturbidimetric assay (Roche Diagnostics Limited, Burgess Hill, UK).

### Outcome definition

For our primary analysis, TB cases were defined as being sputum liquid culture positive for *Mycobacterium tuberculosis* complex (confirmed by GenoType MTBDR*plus* [Hain Lifescience GmbH, Nehren, Germany]). The term ‘TB’ refers to pulmonary TB throughout. Participants with missing CRP or missing or indeterminate culture results were excluded.

### Diagnostic accuracy analysis

This study is reported according to the Standards for Reporting Diagnostic Accuracy Studies. Analyses were conducted in R (version 4.0.2). Supplementary Fig. 1 illustrates our sample size calculation.

We investigated the discriminatory ability of CRP overall, and stratified by HIV status or history of TB, reflecting important risk factors for TB which we hypothesised may modify the diagnostic accuracy of CRP. In further pre-specified analyses, we stratified by age, sex, smoking status, CD4 count, ART, time since previous TB, and indices of disease severity at presentation (presence of anemia, underweight [classified by BMI], TB Score II^14^, and culture days to positivity).

Receiver operating characteristic (ROC) curves were constructed using the ‘pROC’ package.^15^ Confidence intervals of the area under the ROC curve (AUROC) were calculated (DeLong method).^16^ Between-group AUROC were compared by DeLong tests. Correlations between CRP and measures of disease severity were assessed using Spearman rank correlation.

Sensitivities, specificities, and predictive values of CRP were calculated at a primary threshold of 10 mg/L, as proposed previously. We preferred to use a primary threshold higher than the recently recommended WHO threshold for systematic TB screening among PLHIV (>=5 mg/L), since we expected the distribution of CRP values to be higher among our symptomatic population.^17^^,^^18^ In secondary analyses, we used (a) CRP ≥5 mg/L, consistent with WHO guidance for TB screening among PLHIV; (b) the maximum Youden index of the AUROC; and (c) fixed sensitivity at 95% (reflecting the optimum recommended by WHO).^2^^,^^4^

We have previously reported false positive Ultra results in our setting.^12^ We therefore evaluated the potential of CRP to discriminate culture status among Ultra-positive participants. To explore the potential for CRP to aid in diagnosis of people missed by current first-line tests, we also calculated AUROC among Ultra-negative individuals and smear-negative individuals. We stratified analyses by ability to spontaneously expectorate sputum to explore the utility of CRP among people who may be missed by current diagnostics in settings where sputum induction is not available.

### Clinical utility analysis

To assess the potential clinical utility of CRP, we calculated positive and negative predictive values for TB and performed DCA using the ‘rmda’ package.^19^^,^^20^ DCA quantifies ‘net benefit’ as the proportion of true positives minus false positives, weighted by the ‘threshold probability’ for confirmatory testing. The inverse of the threshold probability is the NWT with a confirmatory assay to detect one true positive TB case. In decision curve analysis, the optimal approach is that with highest net benefit across a clinically relevant NWT range. The NWT range is context-specific, influenced by individual and community-level TB-associated costs, and resources available. It is likely to vary across health services and world regions.

We examined the net benefit of CRP (at ≥10 mg/L) to guide confirmatory testing across a NWT range from five to 100 (corresponding to threshold probabilities of one to 20%) and compared this to alternative strategies of offering confirmatory testing to all participants or confirmatory testing to none. Because the primary reference standard was culture-confirmed pulmonary TB, in our study population ‘confirmatory testing for all’ is equivalent to testing all individuals who meet WHO criteria for TB evaluation with a TB culture.

We also compared CRP performance to hypothetical dichotomous biomarkers simulated to meet WHO-recommended optimum (sensitivity 95%; specificity 80%) and minimum (sensitivity 90%; specificity 70%) diagnostic accuracy criteria. We simulated these performance metrics by randomly sampling the relevant proportion of true positive and false positive results in the study population and allocating positive results to sampled individuals, with the remainder allocated as negative.

### Sensitivity analyses

We explored alternative reference standard definitions, using (a) sputum culture- or Ultra-positive (including Ultra-trace) TB; and (b) Ultra alone. We repeated the decision curve analysis with an Ultra reference standard, recognizing that Ultra is frequently the available confirmatory test. We also modelled the potential impact of population TB prevalence (range 5–40%) on predictive value and on DCA results.

### Role of the funding source

The funder had no role in study design; data collection, analysis, or interpretation; writing of the report; or decision to submit for publication. The corresponding authors had full access to all the data and had final responsibility for the decision to submit for publication.

## Results

### Study population

**Fig. 1:**
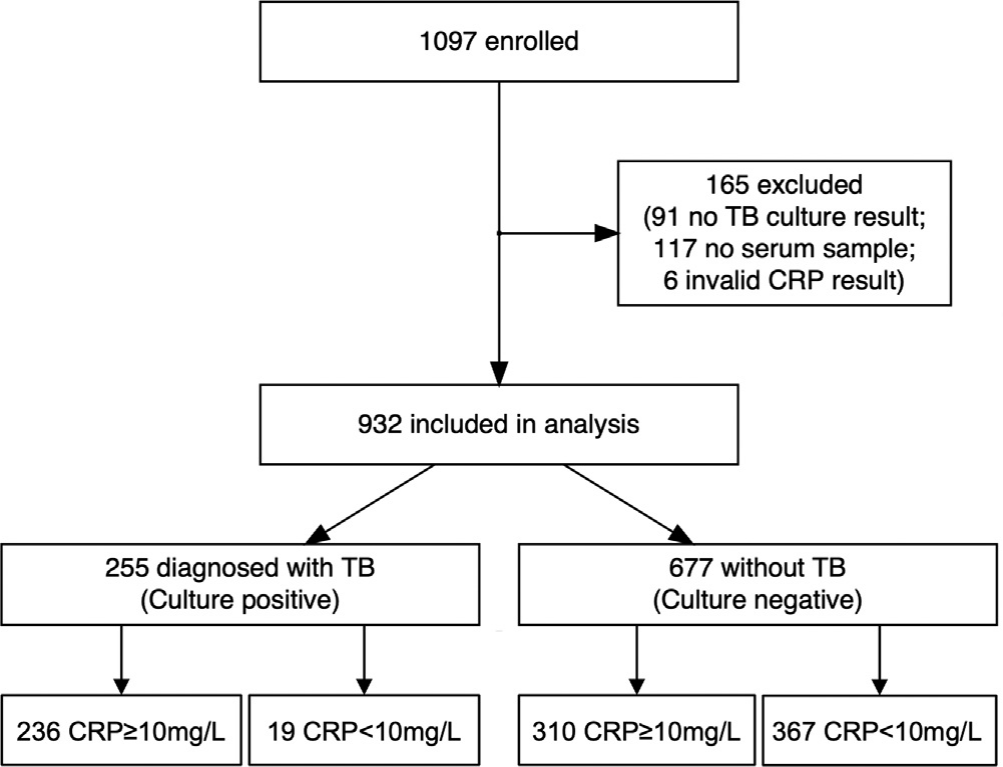
Overview of study cohort.

Between April 2016 and November 2020, 1097 participants were recruited; 932 (85%) had CRP and culture results available and were included (Fig. 1). Characteristics of excluded individuals are in Supplementary Table 1; there were no systematic differences versus those included. Among participants, the median age was 36 years (interquartile range [IQR] 28–47 years). Forty-six percent were female (*n* = 430) and 42% were PLHIV (*n* = 389), of whom 67% were receiving ART (*n* = 259; Table 1). Few PLHIV had a recent CD4 count (*n* = 84 within 3 months of enrolment). Twenty-seven percent of participants were *Mtb.* culture-positive (*n* = 255), among whom the median time to positive culture was eight days (IQR 6–12 days, *n* = 249 with data available).

**Table 1:** Characteristics of study population.

Characteristic	Total	Not TB	TB
**Overall (*n*** **=** **932)**	**932 (100%)**	**677 (100%)**	**255 (100%)**
Scottsdene clinic	497 (53%)	351 (52%)	146 (57%)
Wallacedene clinic	435 (47%)	326 (48%)	109 (43%)
Age (*n* = 932)	36 (28–47)	38 (29–49)	34 (26–43)
Female sex (*n* = 932)	430 (46%)	320 (47%)	110 (43%)
Race (*n* = 932)			
Black	373 (40%)	275 (41%)	98 (38%)
Mixed ancestry	559 (60%)	402 (59%)	157 (62%)
HIV positive (*n* = 924)	389 (42%)	276 (41%)	113 (44%)
Smoking status (*n* = 931)			
Current smoker	531 (57%)	385 (57%)	146 (57%)
Never smoker	257 (28%)	193 (29%)	64 (25%)
Previous smoker	143 (15%)	98 (14%)	45 (18%)
History of previous TB (*n* = 932)	381 (41%)	285 (42%)	96 (38%)
BMI; kg/m² (*n* = 925)	20·3 (18·0–23·9)	21·2 (18·6–24·7)	18·7 (16·8–20·6)
Presence of anemia (*n* = 925)	275 (30%)	139 (21%)	136 (54%)
Presence of cough (*n* = 930)	894 (96%)	642 (95%)	252 (99%)
Presence of fever (*n* = 812)	248 (31%)	176 (30%)	72 (32%)
Presence of weight loss (*n* = 817)	486 (59%)	320 (54%)	166 (73%)
Presence of night sweats (*n* = 930)	664 (71%)	475 (70%)	189 (74%)
AFB smear positive (*n* = 897)	128 (14%)	11 (1.6%)	117 (51%)
Ultra result (*n* = 896)			
Ultra MTB detected (excluding trace)	226 (25%)	16 (2.5%)	210 (85%)
Ultra MTB not detected	636 (71%)	612 (94%)	24 (9.7%)
Ultra MTB trace detected	34 (3.8%)	21 (3.2%)	13 (5.3%)
Able to spontaneously expectorate sputum (*n* = 119)	70 (59%)	47 (55%)	23 (70%)
CRP (*n* = 932)	20 (4–100)	8 (2–55)	99 (56–161)
**Among people living with HIV (*n*** **=** **389)**			
On ART (*n* = 389)	259 (67%)	198 (72%)	61 (54%)
CD4 count; cells/µL (≤3 months) (*n* = 84)	196 (60–355)	232 (67–387)	86 (46–339)
WHO danger signs present (*n* = 363)	35 (9·6%)	16 (6·2%)	19 (18%)

Data are n (%) for categorical variables or median (interquartile range) for continuous data. Anemia was defined as per WHO: hemoglobin <12 g/dL for women and <13 g/dL for men. **Abbreviations:** ART: antiretroviral therapy, BMI: body mass index, CRP: C-reactive protein, WHO: World Health Organization.

### Diagnostic accuracy

**Fig. 2:**
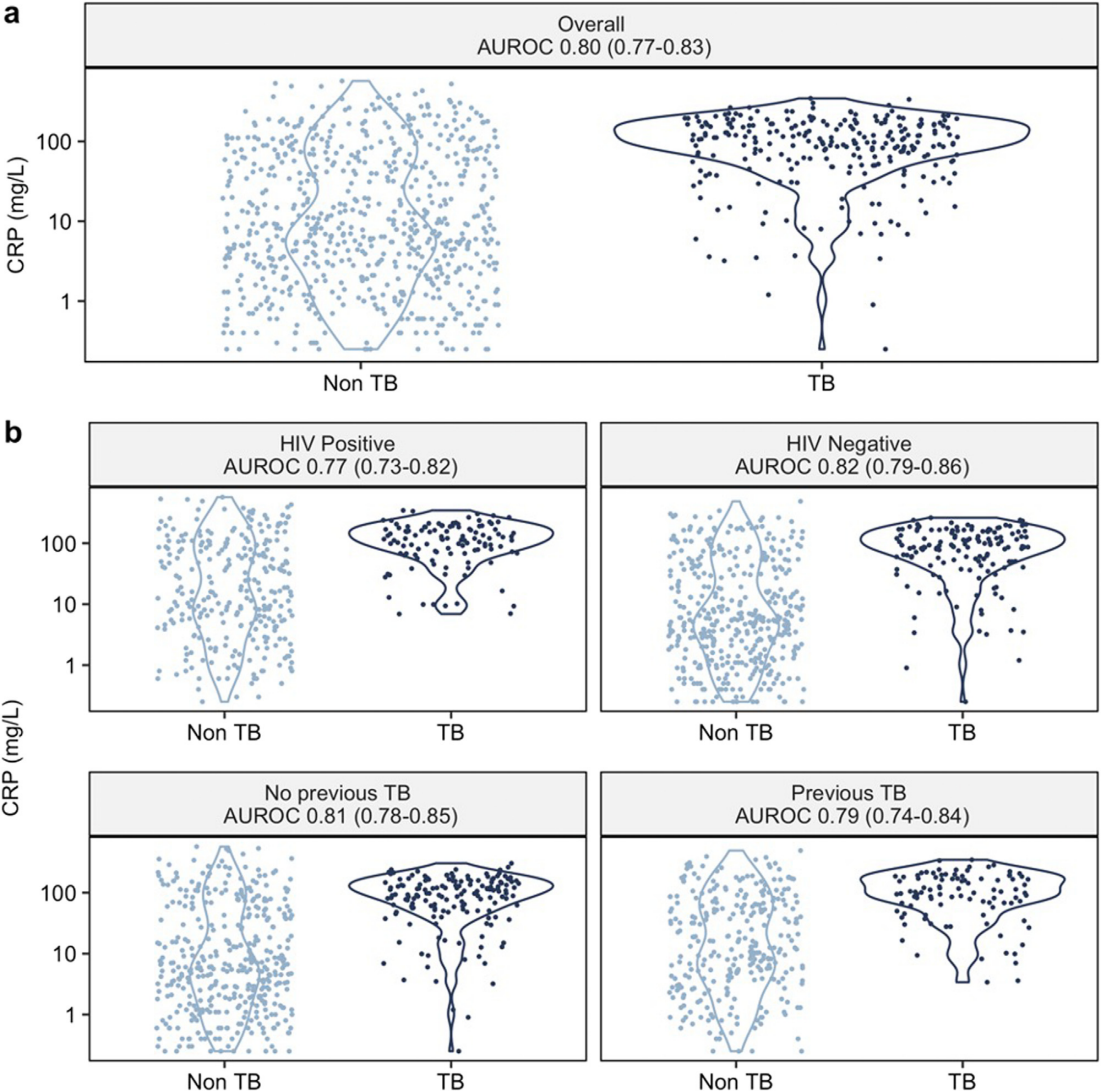
Discriminatory ability of CRP for culture-confirmed pulmonary TB among adults presenting to outpatient care with TB-related symptoms. Area under the receiver operator characteristic curve (AUROC) of CRP to distinguish pulmonary TB versus culture-negative in (a) whole study population and (b) key subgroups of interest; and the distribution of CRP presented on a logarithmic scale. There was no difference in AUROC between HIV positive and HIV negative participants (DeLong test, *p* = 0·1) or among people with a history of previous TB compared to those without (*p* = 0·5).

The overall AUROC of CRP for culture-positive pulmonary TB was 0.80 (95%CI 0.77–0.83). This did not differ by HIV or previous TB status (DeLong test *p* = 0.1 and *p* = 0.5 respectively; Fig. 2). AUROCs did not appear to be affected by age, ethnicity, smoking status, BMI, or time since last TB episode among those with previous TB (*p* ≥ 0.1 across strata of each subgroup; Supplementary Fig. 2). AUROCs were lower amongst people with anemia compared to those without (0·68 [95%CI 0·62–0·75] vs. 0·80 [0·76–0·84]; *p* = 0·003). There was some evidence of lower AUROC among men as compared to women (0·77 [0·73–0·81] and 0·84 [0·80–0·88] respectively; *p* = 0·03).

Using our primary threshold of ≥10 mg/L, 546/932 (59%) of participants had a positive triage test. Sensitivity was 93% (95% CI 89–95%) and specificity was 54% (50–58%), thereby meeting the WHO-recommended minimum 90% sensitivity for a triage test, but not the minimum 70% specificity (Table 2). The numbers needed to test with culture to detect a true pulmonary TB case were 2·3 and 20 among CRP-positive and CRP-negative participants respectively. Using the ≥5 mg/L cut-off, sensitivity was higher (97% [94–98%]), but specificity was only 39% (35–42%). At the maximum Youden index (CRP threshold ≥28 mg/L), CRP approached both minimum WHO target product profile (TPP) criteria, with sensitivity 87% (95%CI 83–91%) and specificity 66% (62–69%; Supplementary Table 2).

**Table 2:** Summary of discriminatory ability of CRP for culture-confirmed pulmonary TB, overall and stratified by key sub-groups of interest.

					CRP ≥10 mg/L					
Subgroup	Cases / N	% Triage positive	AUROC	p	Sensitivity	Specificity	PPV	NPV	NNT (CRP+)	NNT (CRP-)
Overall	255 / 932	59	0·80 (0.77–0.83)	ref	0·93 (0.89–0.95)	0·54 (0.50–0.58)	0·43 (0.39–0.47)	0·95 (0·92–0·97)	2·3 (2·1–2·6)	20·3 (13·2–31·5)
HIV Positive	113 / 389	68	0·77 (0·73–0·82)	ref	0·95 (0·89–0·98)	0·43 (0·37–0·49)	0·41 (0·35–0·47)	0·95 (0·90–0·98)	2·5 (2·1–2·9)	20·8 (9·9–45·1)
HIV Negative	142 / 535	52	0.82 (0.79–0.86)	0.1	0.91 (0.85–0.95)	0.62 (0.57–0.66)	0.46 (0.40–0.52)	0.95 (0.92–0.97)	2.2 (1.9–2.5)	19·7 (11·8–33·4)
No previous TB	159 / 551	55	0·81 (0·78–0·85)	ref	0·92 (0.87–0.95)	0·60 (0·56–0·65)	0·49 (0·43–0·54)	0·95 (0·91–0·97)	2·1 (1·8–2·3)	19·2 (11·5–32·6)
Previous TB	96 / 381	64	0.79 (0·74–0·84)	0.5	0·94 (0·87–0·97)	0·46 (0·40–0·51)	0·37 (0·31–0·43)	0·96 (0·91–0·98)	2·7 (2·3–3·2)	22·7 (10·8–49·1)

Shown as number TB cases / number individuals (N), % of study population with CRP ≥10 mg/L, area under the receiver operator characteristic curve (AUROC), and a p-value from DeLong test for differences across subgroups p. At a threshold of ≥10 mg/L, sensitivity, specificity, positive predictive value (PPV) and negative predictive value (NPV) of CRP are shown with 95% confidence intervals, alongside the number needed to test (NNT) with culture to detect one TB case, among participants triage positive (CRP+) and negative (CRP-) at this threshold.

CRP discriminated culture status among people with positive, non-trace Ultra (AUROC 0·75 [95%CI 0·59–0·91]) and had some discriminatory ability for culture status among participants with trace-detected Ultra (AUROC 0·67 [0·48–0·85]) (Supplementary Fig. 3). At a threshold of 10 mg/L, sensitivity of CRP for culture-confirmed TB among limited to people with positive, non-trace Ultra (*n* = 226) was 97% (93–99%), specificity was 19% (4%–46%). At the same threshold among people with trace-positive Ultra (*n* = 34), sensitivity of CRP was 77% (46-95%) and specificity 48% (26-70%). The AUROC of CRP for discriminating culture-positive pulmonary TB among Ultra-negative participants was 0·66 (95%CI 0·57–0·75; Fig. 4b). Among the subset of participants with a negative smear the AUROC of CRP was 0·77 (95%CI 0·73–0·81).

Data on whether participants were able to spontaneously expectorate sputum were only systematically collected for a subset (those recruited after September 2019, *n* = 127). Here, discriminatory ability of CRP appeared similar among individuals who could not spontaneously produce sputum, compared to those who could, suggesting potential utility for CRP in guiding sputum induction (Supplementary Fig. 3).

Among people with culture-confirmed pulmonary TB, there was strong evidence for an association between higher CRP and higher indices of disease severity (lower BMI, shorter time to culture positivity, lower hemoglobin, and higher TB Score II; all *p* < 0·001; Fig. 4).

### Clinical utility

**Fig. 3:**
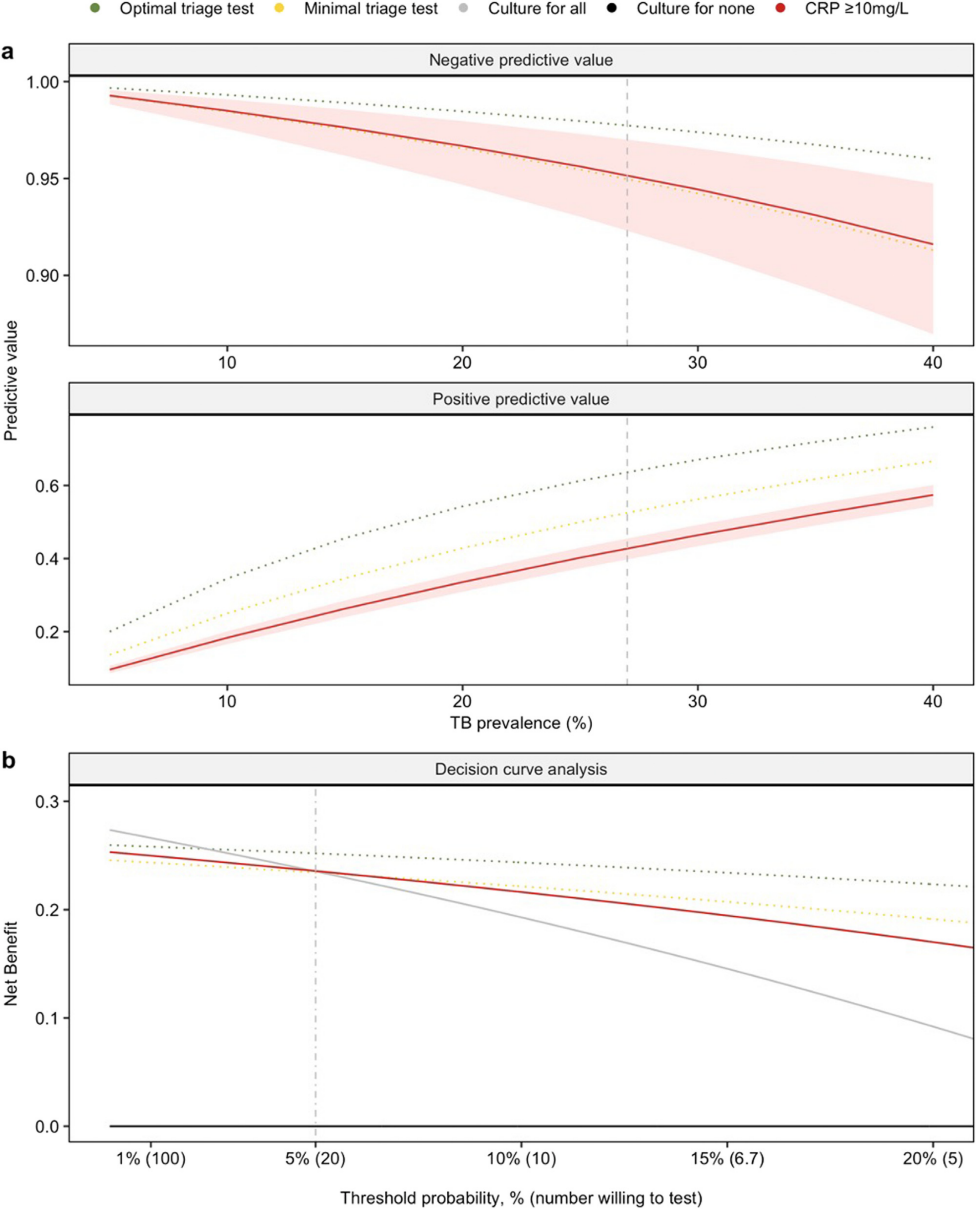
Evaluation of potential clinical utility of CRP as a triage strategy among people presenting with TB-related symptoms Negative and positive predictive values (a) for CRP for culture-confirmed pulmonary TB using a threshold of ≥10 mg/L (red solid line), a hypothetical ‘optimal’ (sensitivity 95%, specificity 80%; green dotted line), and ‘minimal’ (sensitivity 90%, specificity 70%; yellow dotted line) TB triage test as defined by the WHO high-priority target product profile. Reported across a range of TB prevalences (true prevalence in study population indicated by dashed line [27%]). Decision curve analysis (b) comparing the strategies of an ‘optimal’ (green dotted line) or ‘minimal’ (yellow dotted line) triage test or culture for all individuals with CRP ≥10 mg/L (red solid line) to a strategy of culture for all (gray solid line) or none (black solid line) of the participants meeting study inclusion criteria (i.e. presenting with TB-related symptoms, as defined in text). Dot-dash vertical line in panel (b) represents the threshold probability above which CRP confers net benefit over a ‘culture for all’ strategy.

To explore the potential clinical utility of CRP as a TB triage strategy, we first considered the positive and negative predictive values of CRP ≥10 mg/L across a range of TB prevalences, benchmarked against models recreating the optimal and minimal WHO performance characteristics for a triage test. Among adults attending primary care with TB-related symptoms and pulmonary TB prevalence up to 27%, CRP 10 mg/L provided >95% probability that a screened individual does not have pulmonary TB (95%CI 92–97%; Table 2 and Fig. 3a).

**Fig. 4:**
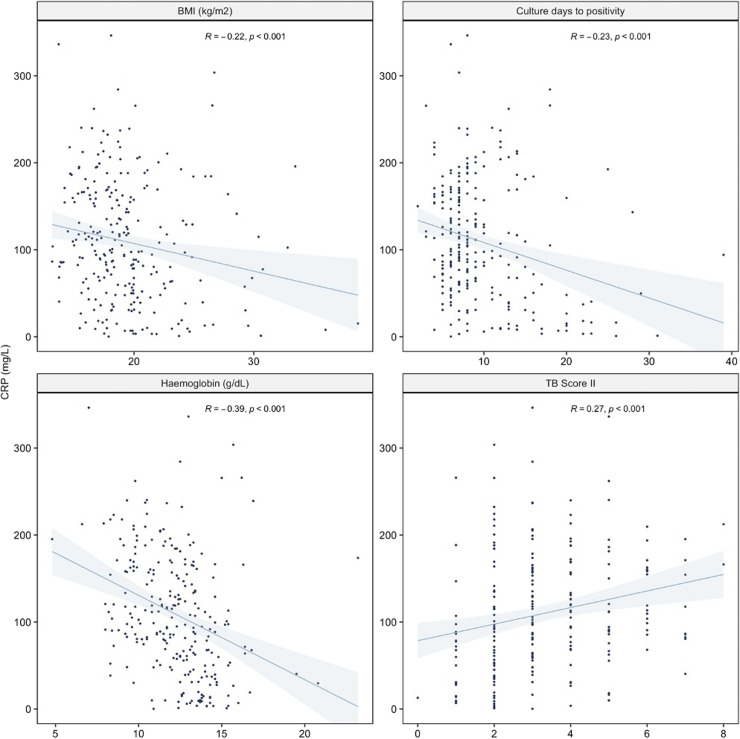
Correlation of CRP with markers of TB severity among culture-confirmed pulmonary TB cases (body mass index ([BMI] culture days to positivity, hemoglobin and TB Score II). TB Score II was only calculated for individuals with complete data for all score components. Spearman rank correlation was calculated.

We then used DCA to further assess the clinical utility of CRP-based triage. At the observed TB prevalence (27%), CRP-based triage demonstrated higher net benefit than a strategy of confirmatory testing for all when the NWT per true positive pulmonary TB case detected was up to 20, reflecting a threshold probability of at least 5% (Fig. 3b). Above this NWT limit, a strategy of confirmatory testing for all performed best. Net benefit for CRP was similar to the simulated WHO minimal triage test across a NWT of seven to 100, whilst the hypothetical optimal triage test had higher net benefit than confirmatory testing for all strategy when the NWT range was up to 33.

### Sensitivity analyses

Sensitivity analyses using a composite microbiological reference standard (Xpert Ultra [including trace] or culture positive pulmonary TB) resulted in reclassification of 37 primary outcome non-TB cases as having TB. Using either this composite reference or an Ultra reference standard, diagnostic accuracy of CRP was similar to the primary analysis (Supplementary Table 5). Results of DCA with an Ultra reference standard were also similar (Supplementary Fig. 5). Compared to culture, an algorithm combining CRP ≥10 mg/L followed by Ultra for triage-positive cases achieved sensitivity 86% (81–90%) and specificity 96% (95%–98%; *n* = 247/896).

Illustrative NWT ranges where CRP, the hypothetical optimal or minimal biomarker had higher net benefit than both confirmatory testing for all and confirmatory testing for none are presented in Supplementary Table 5 across a range of TB prevalences. CRP appears to confer higher net benefit than confirmatory testing for all or none across a wider NWT range at lower TB prevalence.

## Discussion

In this cohort of 932 adults presenting to primary care with symptoms suggestive of TB, CRP (at a pre-defined threshold of ≥10 mg/L) achieved the minimum sensitivity criteria outlined in the WHO TPP for a TB triage test but did not achieve the minimum specificity. Accuracy of CRP was unaffected by HIV or previous TB status, and CRP-based triage demonstrated potential clinical utility to guide confirmatory testing, dependent upon the number of confirmatory tests the health service can perform per TB case detected.

In this population, assuming that the whole study population would usually be systematically tested for pulmonary TB, use of CRP-based triage (≥10 mg/L) would have avoided 382/932 (41%) of confirmatory tests but would miss 18/255 (7%) of pulmonary TB cases. A reduction in the number of confirmatory tests may be desirable in settings where there is insufficient capacity (for example human and analyser capacity) to offer everyone with symptoms a confirmatory test for TB, in order to release capacity for higher risk patients and other case finding interventions (e.g. household contact screening), or where the triage test is cheaper than the confirmatory test, resulting in a cost-saving by using triage to prioritize use of these tests. Decision curve analysis enabled us to quantify the trade-off between correctly detecting true positive and incorrectly identifying false positive cases through triage. Our findings illustrate that the programmatic value of triage is highly dependent on the number of confirmatory tests the health service is willing to perform per case diagnosed. This number is likely to be context-specific, requiring consideration of relevant resource implications and potential costs of missing TB cases. National and international stakeholder engagement are required to further delineate acceptable NWT ranges and support further development of TB triage.

In our setting, CRP-based triage prior to confirmatory testing offered higher net benefit than confirmatory testing for all if the health service is willing to test up to 20 people with a confirmatory test per true positive pulmonary TB case detected (i.e. the threshold probability is set at 5% or higher). If the health service can perform more confirmatory tests than this (i.e. it is deemed that people with less than a 5% likelihood of pulmonary TB should also receive confirmatory testing), a confirmatory test for all strategy (i.e., culture or Ultra for all individuals presenting to clinic and meeting WHO symptom criteria for TB evaluation) is likely to perform better. Benchmarking against WHO TPPs suggested similar clinical utility between CRP and the (hypothetical) minimal-standard triage test, whilst an optimal test may confer additional net benefit across a wider NWT range. The clinical utility of CRP-guided triage is also dependent on TB prevalence: it is likely to be useful across a wider NWT range in settings with lower TB prevalence, while confirmatory testing for all is likely to be preferable when the TB prevalence is high.

While the discriminatory ability of CRP appeared unaffected by HIV status (and ART status among PLHIV), smoking, history of previous TB, age, and BMI, AUROCs were lower among people with anemia and men. Male sex and anemia are both associated with later presentation and more severe pulmonary TB.^21^^,^^22^ Our findings of lower discriminatory ability of CRP for TB among these subgroups may reflect lower specificity among more unwell participants, since CRP is increased in response to a variety of infectious or inflammatory stimuli and correlates with degree of inflammation. CRP is associated with multiple indices of disease severity among pulmonary TB cases in our study, suggesting that CRP-based triage is more likely to detect people who are more likely to transmit *Mtb*. and experience poor outcomes. These individuals would also be more likely to have been offered a TB test in routine care based on clinical judgement if no systematic triage/diagnostic algorithm was place. CRP offered some discrimination for culture status among participants with positive Ultra. This suggests a potential role for CRP in resolving false positive Ultra results: a positive CRP together with a positive, non-trace Ultra has high sensitivity for culture-confirmed pulmonary TB, together with a trace-positive Ultra sensitivity was moderate. CRP also demonstrated some discriminatory ability for culture status among Ultra and smear-negative individuals, and, in a subgroup analysis, those who could not spontaneously expectorate sputum, suggesting CRP may be useful to direct further evaluation among people with negative first-line test results.

Our study is the first, to our knowledge, to report the diagnostic accuracy of CRP for pulmonary TB in a large, consecutive cohort of outpatients presenting with symptoms compatible with TB and mixed HIV status; and is the first to benchmark CRP-based triage against an Ultra-reference standard. We report higher AUROCs among the HIV-negative subgroup than a recent multi-center case-control study, providing strong evidence of utility irrespective of HIV status. In the previous study, post-hoc analyses suggested lower accuracy in southeast Asian settings (where participants were recruited at referral centres) compared to populations in southern Africa.^10^ Future studies should further assess CRP diagnostic accuracy in additional settings, including those with lower TB and/or HIV prevalence, consider head to head comparison of CRP against the WHO symptom screen and consider implementation in health facilities settings where confirmatory testing requires onward referral. We used a robust culture-based reference standard for pulmonary TB (albeit with one, rather than the optimal of two, sputum cultures); with sputum induction for participants unable to produce adequate samples spontaneously. Future studies should evaluate performance of CRP for extra-pulmonary TB.

There are several limitations to this work. First, CRP was measured retrospectively on stored samples. While we propose that CRP is a promising tool for point-of-care use, prospective validation using such a platform is required. Previous studies have, however, demonstrated stability of serum CRP over long-term storage and validated quantitative point-of-care CRP against laboratory-based assays.^23^^,^^24^ Second, we are unable to characterize the ‘non-TB’ diagnoses which may also include individuals with extra-pulmonary TB. Specifically, we do not have follow-up data to determine if any of these cases went on to receive TB treatment soon after their assessment in the study. This reflects programmatic conditions. Third, among included PLHIV, few had a recent CD4 count, reflecting increasingly infrequent use of CD4 in the era of ‘test and treat’. This limited evaluation of the discriminatory ability of CRP across degrees of immunosuppression; previous data suggest sensitivity of CRP for TB may be higher, and specificity lower, among people with lower CD4 counts.^17^ Fourth, it is increasingly recognised that TB symptoms may be minimal: diagnostic algorithms that rely on individuals reporting symptoms as an initial gateway may miss cases. Here, we applied a relatively broad symptom screen, however future triage test development should consider the impact of symptom-based ‘pre-screening’ on triage performance. Finally, we used complete case analysis, excluding participants with missing CRP results or *Mtb*. culture results. There was, however, no evidence of systematic differences between included and excluded individuals, suggesting low risk of selection bias.

## Conclusion

CRP-based triage among symptomatic adults in a high TB and HIV-burden setting achieved the minimum sensitivity, but not the minimum specificity, required by WHO diagnostic accuracy targets . Nevertheless, CRP has many desirable operational characteristics and demonstrated clinical utility. Additional value may be provided through inclusion of CRP in a clinical prediction model, together with other demographic or clinical predictors of risk, or in combination with other biomarkers.^25^ Future interventional and health economic studies are required to further evaluate the potential programmatic role of CRP-based triage. Our analyses provide a framework to move beyond diagnostic accuracy measures and assess the clinical utility of triage strategies for TB in future studies.

## Data sharing

The de-identified patient data, study protocol, informed consent form, and datasets generated and/or analysed are available from BWPR and CJC to researchers who provide a methodologically sound proposal and after signing a data access agreement.

## Contributors

GT and MN conceived and designed the study. BWPR, TM, ZP, GN, and HM were responsible for data collection. CJC and RKG analysed the data with input from BWPR, GT, and MN. CJC produced the figures. CJC and RKG drafted the manuscript. All authors had access to the data, contributed to data interpretation, critically reviewed the manuscript and approved the decision to submit for publication.

## Funding

This work was supported by the South African Medical Research Council (SAMRC Flagship Project MRC-RFA-IFSP-01-2013 to GT) and a Royal Society Newton Advanced fellowship (NA-150-202 to GT). GT acknowledges funding from the EDCTP2 program supported by the European Union (RIA2018D-2509, PreFIT; RIA2018D-2493, SeroSelectTB; RIA2020I-3305, CAGE-TB) and the National Institutes of Health (D43TW010350; U01AI152087; U54EB027049; R01AI136894). MN is funded by the Wellcome Trust (207511/Z/17/Z), and by National Institute of Health Research (NIHR) Biomedical Research Funding to UCL and UCLH. CJC, IA, and RKG report funding from the NIHR (DRF-2018-11-ST2-004 to RKG; NF-SI-0616-10037 to IA). RKG is also supported by the Royal College of Physicians (James Maxwell Grant Prophit Fellowship). CJC is supported by Wellcome Trust (203905/Z/16/Z). Additionally, Cepheid gave in-kind cartridges and equipment donations to the clinical study (BAR-TB) from which the specimens in this manuscript were derived.

## Declaration of Competing Interest

Other than research funding acknowledged above, the authors do not report any conflicts of interest. This paper presents independent research supported by the NIHR. The views expressed are those of the author(s) and not necessarily those of the UK National Health Service, the NIHR or the UK Department of Health and Social Care.

## Appendix Supplementary materials

Image, application 1
